# Implementing referral guidelines: lessons from a negative outcome cluster randomised factorial trial in general practice

**DOI:** 10.1186/1471-2296-7-65

**Published:** 2006-11-02

**Authors:** Moyez Jiwa, Paul Skinner, Akinoso Olujimi Coker, Lindsey Shaw, Michael J Campbell, Joanne Thompson

**Affiliations:** 1 Professor of Primary Care, Curtin University of Technology, GPO Box U1987, Perth, WA 6845, Australia; 2 Northern General Hospital, Sheffield, UK; 3 Doncaster Royal Infirmary, Doncaster, UK; 4 Institute of General Practice, University of Sheffield, Sheffield, UK

## Abstract

**Background:**

Few patients with lower bowel symptoms who consult their general practitioner need a specialist opinion. However data from referred patients suggest that those who are referred would benefit from detailed assessment before referral.

**Methods:**

A cluster randomised factorial trial. 44 general practices in North Trent, UK. Practices were offered either an electronic interactive referral *pro forma*, an educational outreach visit by a local colorectal surgeon, both or neither. The main outcome measure was the proportion of cases with severe diverticular disease, cancer or precancerous lesions and inflammatory bowel disease in those referred by each group. A secondary outcome was a referral letter quality score. Semi-structured interviews were conducted to identify key themes relating to the use of the software

**Results:**

From 150 invitations, 44 practices were recruited with a total list size of 265,707. There were 716 consecutive referrals recorded over a six-month period, for which a diagnosis was available for 514. In the combined software arms 14% (37/261) had significant pathology, compared with 19% (49/253) in the non-software arms, relative risk 0.73 (95% CI: 0.46 to 1.15). In the combined educational outreach arms 15% (38/258) had significant pathology compared with 19% (48/256) in the non-educational arms, relative risk 0.79 (95% CI: 0.50 to 1.24). *Pro forma *practices documented better assessment of patients at referral.

**Conclusion:**

There was a lack of evidence that either intervention increased the proportion of patients with organic pathology among those referred. The interactive software did improve the amount of information relayed in referral letters although we were unable to confirm if this made a significant difference to patients or their health care providers. The potential value of either intervention may have been diminished by their limited uptake within the context of a cluster randomised clinical trial. A number of lessons were learned in this trial of novel innovations.

## Background

Few patients with diarrhoea or rectal bleeding consult a general practitioner and fewer still need referral for specialist advice [1]. In the UK guidelines describe patients who need to be referred urgently [2]. Here general practitioners are audited on their efforts to identify such cases with reference to guidelines published by the Department of Health [3]. It has been reported that over ninety percent of patients with colorectal cancer satisfy the published criteria for urgent referral and yet most patients who merit an urgent referral do not have cancer [4]. It has also been suggested that delayed diagnosis may result in part for a failure to elicit the relevant history or perform the pertinent examination of symptomatic patients [5]. Therefore the detailed assessment of patients with colorectal symptoms is considered to be vital when choosing which patients to refer in order to achieve a timely diagnosis.

We report a trial in which an electronic interactive referral *pro forma *prompting the assessment of patients with lower bowel symptoms was introduced in general practice as part of the workflow. It was anticipated that practitioners would learn which signs and symptoms were important and which patients required urgent referral. Adult learning theories predict that when practitioners are offered guidance at the time of making a decision, 'learning' or a *'relatively permanent change to the frequency of actions brought about by instruction or reinforced practice' *will take place [6,7]. Empirical evidence from Patel and Kaufman indicate that the integration of software systems into clinical settings fundamentally change not only how physicians view their daily work practice but also the very process of medical reasoning itself [8]. Mugford and colleagues identified 36 published studies of interventions of feedback of information, concluding that information feedback was most likely to influence clinical practice if the information was presented close to the time of decision-making and the clinicians had previously agreed to review their practice [9].

Data from the US suggests that in-office education of primary care physicians may be effective in improving awareness of significant clinical features [10]. Similarly a Cochrane review concluded that educational outreach visits, may be a promising approach to modifying health professional behaviour [11]. Presentations by invited clinical experts are often feature in educational events aimed at general practitioners. Thus we also included a visit by a colorectal surgeon as part of an educational outreach programme as a second intervention. The research question is whether the introduction of an electronic interactive referral *pro forma *or educational outreach visits by a colorectal surgeon to general practice can alter the case mix of patients referred to lower bowel specialists.

## Methods

### Recruitment

Practices were recruited from Doncaster and Sheffield. Ethical review was by the North Sheffield Research Ethics Committee. Referrals to colorectal surgeons in this region were not required on any existing *pro forma*. A total of 150 practices were offered the opportunity to participate and 44 practices were recruited (29%) from August to December 2003 (see Additional File 1).

### Development of software intervention

**Figure 1:**
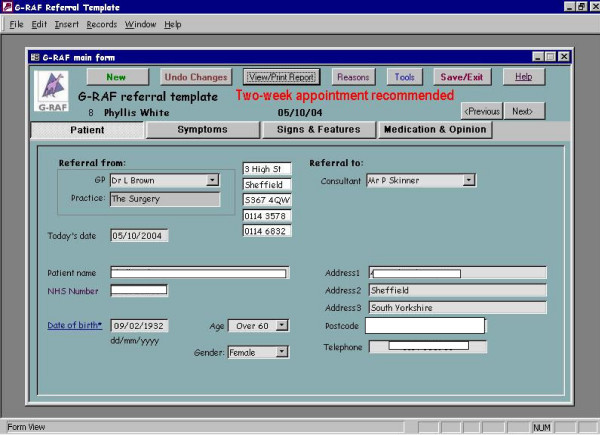
Fields on the GRAF software, opening page.

**Figure 2:**
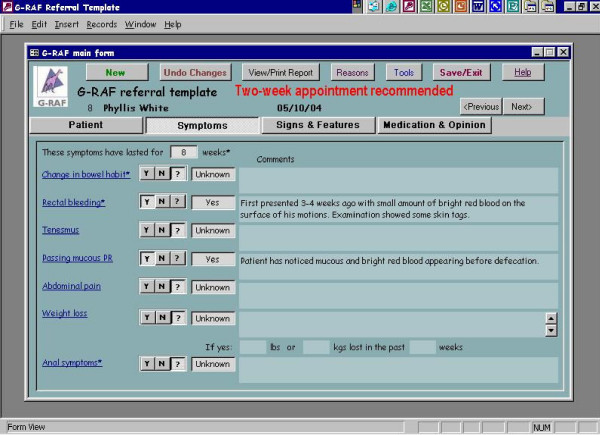
Fields on the GRAF software, screen 2.

**Figure 3:**
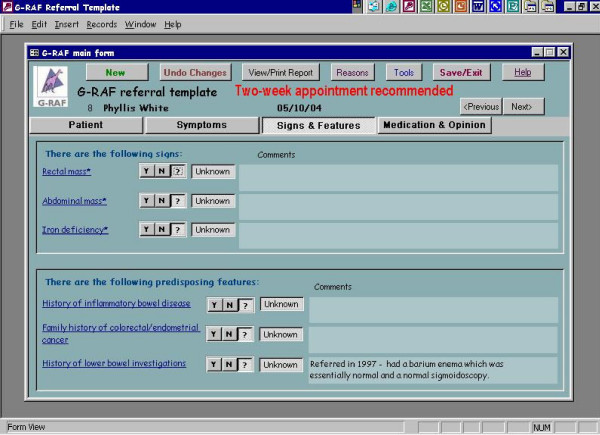
Fields on the GRAF software, screen 3.

**Figure 4:**
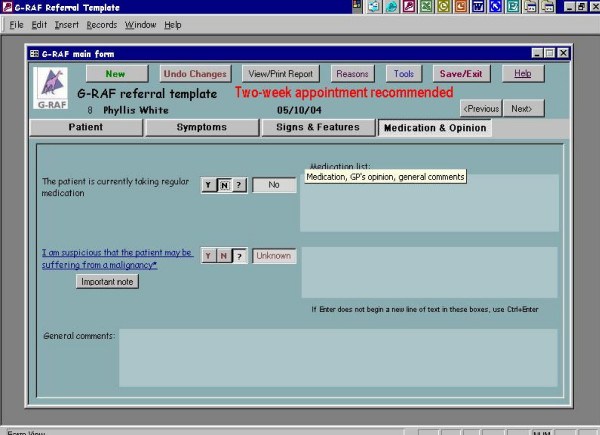
Fields on the GRAF software, screen 4.

We developed and piloted an interactive electronic *pro forma *for processing referrals to colorectal surgeons (**G**eneral Practice **R**eferral **A**ssessment **F**acilitator or G-RAF). The interactive *pro forma *requested information on drop down menus for fifteen clinical signs and symptoms previously identified by GPs and colorectal surgeons as those of significant colorectal disease [12]. Once the clinical data were entered on the *pro forma *the interactive software offered the practitioner guidance on which cases needed urgent referral with reference to current UK Department of Health guidelines [3]. Once clinical data were entered a referral letter was automatically produced seeking an appropriate appointment at a hospital clinic. (See screen grabs in Figure 1, 2, 3, 4). It was not possible to merge G-RAF with the practice software, as there were a plethora of different clinical software systems deployed at local practices. A member of the project team who offered technical support throughout the project trained GPs in the use of G-RAF on a one-to-one basis at their practice. The software was installed in the relevant practices by March 2004 and was available for the duration of the project.

### Educational outreach (EO)

A colorectal surgeon delivered short educational sessions. The design of the package was modified following pilot presentations to small numbers of practitioners and subsequently tailored to the needs of the target audience as expressed in the feedback to the team. The outreach visits were designed to reflect the model for content developed by Soumerai and Avron and incorporated general principles of education and behaviour change in the training sessions [13]. During the 45 minute EO meeting, the presenter summarised the features of significant organic colorectal disease and encouraged questions. The published guidelines and the potential for the improvement to the management of patients were emphasised [3]. This intervention was delivered from December 2003 to March 2004.

### Design and sample size calculations

The design is a 2 × 2 factorial design, clustered by practice. The fours arms are: no educational outreach or software; educational outreach only; interactive *pro forma *only; educational outreach and interactive *pro forma*. This design enables one to estimate the effect of the software independently of the effect of the educational outreach. We assume *a priori *that there is no interaction between the two interventions, which is reasonable since they have different modalities. Having consented to take part, the practices were randomised to one of the four groups, stratified by median practice size. A computer program was used to allocate practices at random, using a fixed block size to ensure approximately equal numbers in the four arms. A pilot study suggested that the proportion of subjects referred by GPs who had 'significant' pathology was approximately 0.14 and these values are assumed for the 'no educational outreach-no software' group of the proposed trial. These pathologies were listed by consensus among local specialists and included: cancer; large polyps (≥3 cm); moderate to severe diverticular disease, and inflammatory bowel disease. We anticipated that the intervention would be valuable if it increased the proportion to 0.25 (i.e. a relative risk of about 2). A non-clustered trial would require about 630 referrals to achieve 80% power at two-sided 5% significance. The interclass correlation coefficient from previous data was calculated as 0.03 [14]. On average, each GP refers six patients per year. Therefore the Design Effect is 1.15 and the required number of referrals is 725. With approximately three GPs per practice, this suggests that around 40 practices were required.

### Statistical methods

A research associate familiar with medical terminology and with reference to a clinician when necessary collected data for the trial at the hospitals. A medical practitioner and researcher both blind to the arm of the study in which the referral was made coded the outcomes of the study independently. Differences in coding were resolved by a colorectal surgeon. The quality of the referrals was assessed by scoring each referral letter with one point for each of fifteen items considered relevant to a colorectal surgical referral as shown in Table 1[12]. This proxy marker for the quality of referral letters was known as the 'Assessment Score'.

**Table 1:** Clinical data that should be relayed to the specialist.

Duration of symptoms
History of change in bowel habit
History of rectal bleeding
History of tenesmus
History of passing mucous per rectum
History of abdominal pain
History of weight loss or patient's weight.
History of peri-anal symptoms e.g. itch or pain
Rectal mass or results of rectal examination
Abdominal mass or results of abdominal examination
Iron deficiency anaemia or results of full blood count
History of inflammatory bowel disease
Relevant family history
History of lower bowel investigations or existing colorectal conditions
General practitioner's opinion as to likely diagnosis

The outcome measures were: the proportion of cases with significant colorectal organic pathology referred in each arm of the study, an 'Assessment Score' of the quality of the referral letters reflecting the number of signs, symptoms and other data recorded in the referral letters and the proportion of cases from each arm referred on the appropriate pathway. The relative risk is an appropriate outcome in prospective studies, and we used a method described recently by Zou based on a modified Poisson regression, which also allowed for clustering of the data [15]. The 'Assessment Score' was found to be skewed. Therefore a square root transformation was used to give a more symmetrical distribution. The data were collected at the hospitals receiving the referrals.

### Interviews

Six months after the referral *pro forma *was introduced practitioners using the software were asked to complete a questionnaire relating to their use of the software. We then conducted a series of in depth interviews on selected GPs on the basis of age; gender and self reported use of software. The practitioners were aged from 30 to 60 years. Interviews were audiotaped and transcribed. The transcripts from the groups were analysed by two researchers working independently. Analysis was by the framework method drawing on *a priori *issues and questions derived from the aims of the study as well as issues raised by the respondents themselves and views and experiences that recurred in the data [16]. Sampling was terminated once saturation of themes was considered to have been achieved. The analysis followed the prescribed steps, including: familiarisation, indexing, charting, mapping and interpretation. Following analysis interviewees were asked to review a summary of the themes and to offer any further ideas on reflection. We present key themes only in this report.

## Results

Practice characteristics are shown in Table 2. The 44 practices included 180 general practitioners serving 265,707 patients. There were 716 referrals over a six-month period from April to September 2004. Referral letters were traced for 531 cases, diagnosis was available in 514 cases and pathway of referral was known in 504 cases. The records 'lost' in the hospital administrative systems were evenly spread between all four groups and therefore we had no reason to suppose there was any systematic bias in the data collected.

**Table 2:** Practices by intervention group

	**Software**	**EO & Software**	**EO**	**Control**
*Practices*	12	9	11	12
*List size (mean)*	5894	6263	6599	5001
*Mean WTE GPs*	3.7	3.9	4	3.1

### Uptake of the interventions

**Figure 5:**
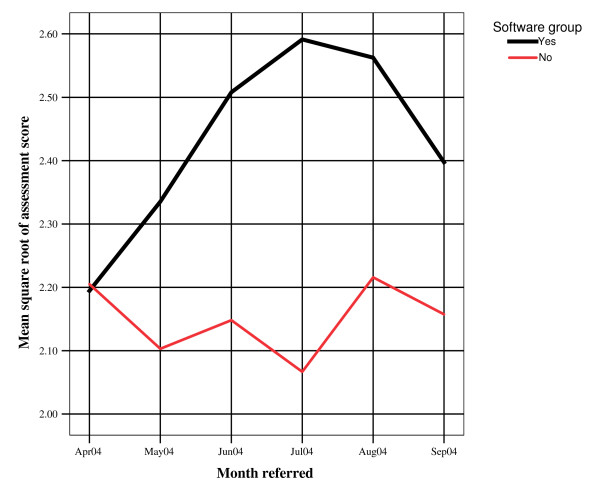
Assessment of patients in documented referral letters. Software practices vs. others

On average 18.1% of referrals in the software arm were processed on the software. Figure 5 shows the Assessment Score for the software practices improved through time and began to dip towards the end of the trial mirroring the attrition in the use of the software. Just under sixty percent (47/80) of practitioners attended the educational outreach visit by the surgeon. All were sent videotaped presentations by the surgeon covering the same material.

### Outcome measures

Table 3 shows there was no statistical difference in the proportion of cases with significant colorectal pathology for either intervention, with neither intervention performing better than the control, and indeed the point estimates suggesting the interventions performing worse. The 95% confidence intervals suggest that we can rule out a 25% improvement in relative risk, which with a 14% baseline rate, translates as about a 4% absolute improvement. The interventions could give as much a 7% lower absolute percentage in referrals with significant pathology. Analysis by cancer diagnosis alone failed to detect any significant trend with cancers referred by each group: Software practices (5 Colorectal cancers (CRCs) + 2 other cancers), Educational Outreach group (4 CRCs + 2 others), both interventions (1 CRC + 3 others), control group (4 CRCs and 4 others). Table 4 shows that practitioners in the software arm wrote referral letters, which had more information than the controls, but educational outreach did not improve the quality of the letters.

**Table 3:** Proportion of cases with significant pathology referred by each arm of the trial

	**'Significant' pathology**	**Total**	**%**	Relative Risk (95%CI)* P-value
*Software*	37	261	14.2%	0.73 (0.46 to 1.15) P = 0.18
*Non-software*	49	253	19.4%	

*Educational Outreach*	38	258	14.7%	0.79 (0.50 to 1.24) P = 0.30
*Non-Educational Outreach*	48	256	18.8%	

*Adjusted for clustering by practice

**Table 4:** Assessment score of letters for patients referred by each arm of the trial (square root)

	**Assessment Score**	**Mean difference (95% CI)**	**P-value**
*Software*	2.4	0.3 (0.17 to 0.42)	<0.001
*Non-SW*	2.1		

*EO*	2.34	0.08 (-.04 to 0.2)	0.18
*Non-EO*	2.25		

### Interviews

We recorded a 60% response rate to the questionnaires about the use of the software. Eight practitioners were interviewed about their use of the software before saturation of themes was achieved. Two key themes were identified relating to poor adoption of the software in the interviews with users these were:

#### i) Duplication of effort

Typically one GP expressed concern that the pro forma created an additional task in the process:

"I suspect there are a lot of GPs ... that are not interested in that they just want to make the referral. It is very much quicker for me to dictate a letter to my secretary and send it – a lot less effort. GRAF is installed on all the computers here and I guess there are only two of us that use it on a regular basis."

#### ii) Doubts about the software

The final question in the pro forma invites the GP to say if he/she thinks the patients' symptoms are those of cancer. Three choices were offered on the drop down menu: 'yes', 'no' and 'don't know'. If the practitioner answered 'yes' the referral was processed as an urgent referral. If they answered 'no' and the clinical details, with reference to the guidelines suggested otherwise, the program warned the practitioner that they had chosen to over rule guideline recommendations. The software was programmed to respond to the answer 'don't know' by recommending the most appropriate pathway based on the clinical details entered with reference to the published guidelines. Commenting on this function one respondent expressed a frequently voiced misgiving about GRAF.

*"There's a lot of people that you don't think have got cancer, but ... you're not going to be sure. You don't know they definitely haven't got cancer, and that's partly why you refer most people who've got bowel symptoms, especially, you know, there's a bit of you might be worried that there may be a malignancy. So quite often you put the "Don't know", and if they all then become two-week wait *(i.e. urgent referrals), *then I think a lot of people are getting two-week waits that probably shouldn't get two-week waits. I don't want it *(the hospital clinic) *to sort of get overloaded with urgent two-week appointments that aren't really appropriate."*

## Discussion

We are disappointed to report little or no benefit from the outreach visits or from the adoption of the *pro forma *software, which was limited to one in five referrals from the software practices. The purpose of the electronic innovation was to increase consultation of guidelines. The design of the *pro forma *as a workflow innovation was to enable practitioners to complete a task, namely write a referral letter. The data suggests there was minimal enthusiasm for the letter writing facility offered in the program. A previous study similarly concluded that despite their apparent benefits computerised referral *pro forma *systems are unpopular on the face of it because they transferred the administrative burden of the referral process from clerks and secretaries to clinicians [17]. Similarly experience with web-based programmes in primary care has been disappointing [18]. It is possible, perhaps probable, that we did not offer interventions that were acceptable in practice rather than cast doubt on the potential value of electronic referral *pro formas *and outreach visits per se. The fact that a reasonable number of practices were recruited but failed to participate echoes the experience of others who similarly reported disappointing results from studies in which practitioners remained ambivalent toward the proposed innovations [19]. We suspect however that more valuable lessons can be learned from this negative experiment in addition to the difficulty of conducting large clinical trials in general practice.

Firstly our electronic innovation and to some extent the outreach visits were aimed at influencing medical decision making by focusing on the task of documenting and relaying medical information, that is on the medical record. We hypothesised, with reference to Adult learning theory that as clinicians were led to be more comprehensive in the data relayed to specialists that their clinical acumen would increase. We sought evidence for this from the greater number of referrals of patients with 'significant' bowel pathology. It is possible that the designation of 'significant' pathologies, those that were thought to be the root cause of the symptoms, including severe diverticular disease, identified the 'wrong' group of disorders and so we were unable to detect any 'significant' improvement in diagnostic acumen. In addition applying this innovation to other professional groups, for example practice nurses or involving the patient directly in documenting the clinical details may have yielded different results. We believe the software did identify the patients who met the referral criteria for urgent referral insofar as the software was programmed with the relevant algorithms. However this presupposes that the clinician systematically elicited the relevant clinical features for all symptomatic patients referred or otherwise.

Secondly we did not formally evaluate whether the educational visits resulted in any improvement in knowledge or change in practitioner attitude and so could not confidently confirm that the visits were effective. Thirdly the literature on the purpose of medical records suggests that the document genres evolve as their purpose and content evolves [20]. The purpose of the 'referral letter', at the time of the project and as stated in the interviews was to seek a specialist opinion, not as our innovation was fashioned as a prompt for the detailed clinical assessment of symptomatic patients and a way to consult guidelines. The role of the referral letter did not 'evolve' to this purpose.

Finally it was apparent in this trial that general practitioners were uncomfortable with challenges to their clinical judgement. It is such attitudes that might hold the key to the implementation of guidelines more than, or in addition to, a technical fix-it or educational exchange. A key concept in the relation to the adoption of complex technologies is that they involve groups and networks of people [21]. Technology both supports and shapes social practices such as sending a patient for a specialist opinion. In such communities, knowledge " rides along the rails laid by shared practice" [22]. Where practices are canonical (agreed, shared) knowledge flows readily; where practices are noncanonical (innovative, challenging), knowledge may 'stick' rather than flow [21]. It was apparent in our data that general practitioners sometimes had very different ideas about what constitutes an 'urgent' referral and that this further cast doubt on the value of the software for them. The result was a rejection of the innovation and by default the guidelines.

## Conclusion

Along with other negative IT projects we did not take account of how our innovation was to be used in practice and perhaps more importantly how its use might threaten established professional identities and work practices [23]. We need a clearer understanding of what clinicians do in relation to specialist referrals and at the 'macro' level what organisational routines, scripts and structures support them. This trial ignored the 'messiness' and ad-hoc nature of clinical work. We support those who argue that this is not a minor issue, which will be tidied up if only clinicians learn to adopt electronic innovations more consistently and rationally. We echo Greenhalgh and colleagues in their plea for further research into the spread and sustainability of technology based innovations in health care [23]. We also recommend that any future trial of this approach in general practice is more closely integrated with the electronic health record.

## Competing interests

The author(s) declare that they have no competing interests.

## Authors' contributions

All authors contributed equally to this work

## Pre-publication history

The pre-publication history for this paper can be accessed here:



## Supplementary Material

Additional file 1 Consort flow chart Click here for file
