# Supplementary figures and images for: Implementing referral guidelines: lessons from a negative outcome cluster randomised factorial trial in general practice
Source: BMC Fam Pract. 2006 Nov 2;7:65. doi: 10.1186/1471-2296-7-65 (PMC1635053; doi:10.1186/1471-2296-7-65)

## The Consort Flowchart

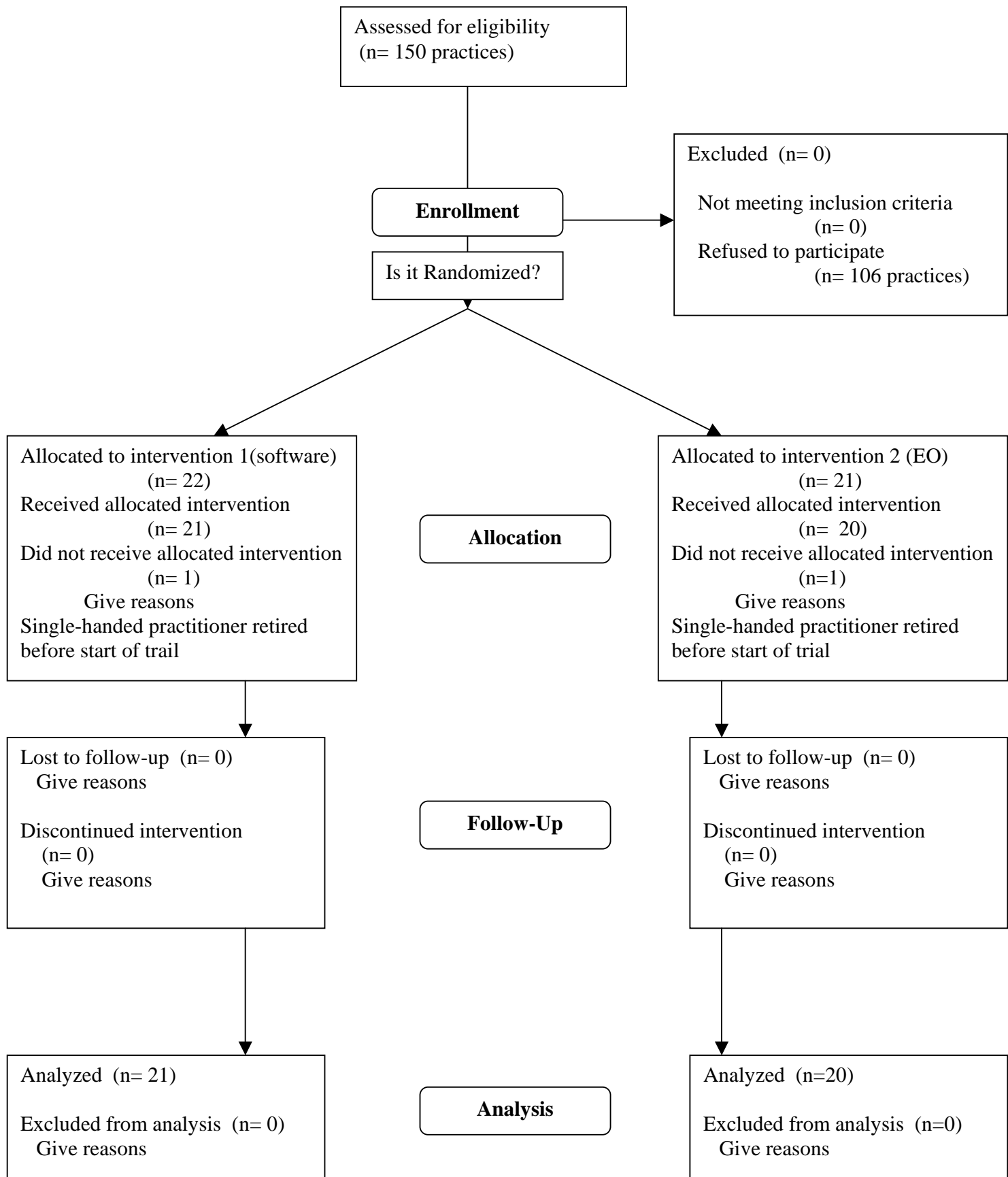

Supplement: Additional file 1 — Consort flow chart [file 1471-2296-7-65-S1.pdf]
